# Introduction to Personalized Medicine in Diabetes Mellitus

**DOI:** 10.5041/RMMJ.10136

**Published:** 2014-01-21

**Authors:** Harry S. Glauber, Naphtali Rishe, Eddy Karnieli

**Affiliations:** 1 Department of Endocrinology, Northwest Permanente, Portland, Oregon, USA;; 2 Florida International University, Miami, Florida, USA;; 3 Institute of Endocrinology, Diabetes and Metabolism, Rambam Medical Center, Haifa, Israel and; 4 Galil Center for Telemedicine, Medical Informatics and Personalized Medicine, RB Rappaport Faculty of Medicine, Technion – Israel Institute of Technology, Haifa, Israel

**Keywords:** Diabetes mellitus, personalized medicine, pharmacogenomics, prediction of diabetes complications, prediction of diabetes mellitus

## Abstract

The world is facing an epidemic rise in diabetes mellitus (DM) incidence, which is challenging health funders, health systems, clinicians, and patients to understand and respond to a flood of research and knowledge. Evidence-based guidelines provide uniform management recommendations for “average” patients that rarely take into account individual variation in susceptibility to DM, to its complications, and responses to pharmacological and lifestyle interventions. Personalized medicine combines bioinformatics with genomic, proteomic, metabolomic, pharmacogenomic (“omics”) and other new technologies to explore pathophysiology and to characterize more precisely an individual’s risk for disease, as well as response to interventions. In this review we will introduce readers to personalized medicine as applied to DM, in particular the use of clinical, genetic, metabolic, and other markers of risk for DM and its chronic microvascular and macrovascular complications, as well as insights into variations in response to and tolerance of commonly used medications, dietary changes, and exercise. These advances in “omic” information and techniques also provide clues to potential pathophysiological mechanisms underlying DM and its complications.

## INTRODUCTION

Recent decades have seen a dramatic worldwide increase in the incidence and prevalence of diabetes mellitus (DM), particularly of type 2 DM.^1^ The potential impact of DM on health, health care costs, productivity, and life expectancy in the upcoming years will be enormous. Contemporaneously, there has been substantial progress in a wide range of treatments for DM and for its chronic complications, leading to improved life expectancy.^2^,^3^ Expert bodies now regularly publish “standards of care” and recommendations addressing all aspects of DM management.^4^–^6^ Such documents address the care needs of the average or typical patient with DM, based largely on the findings of studies advancing our understanding of the pathophysiology of DM and its complications, as well as randomized control treatment trials. Optimal therapy of DM requires potentially complex measures to control hyperglycemia, prevent hypoglycemia, and to address risk factors for a range of diabetic complications. The caregiver has also to be aware of the patient’s social, cultural, and eco-system components (environmental components within the closed community—like housing and neighborhood). Many patients are unable to reach treatment goals due to the difficulty, complexity, and costs of treatment.^7^ Recognizing the wide range of patients with DM, recent guidelines now stress the need to personalize DM management goals and treatments.^8^

In the face of the “diabetes tsunami”^9^ the gap between knowledge derived from basic scientific and clinical research, including newly recognized molecular mechanisms and updated medical management guidelines and their use at the bedside or point of care by practitioners, is growing. Developing strategies and tools to bridge this knowledge and implementation gap is increasingly urgent as medically relevant and novel scientific discoveries can now be applied to assess risk factors at the genomic level for chronic diseases like cancer and DM, as well as the sensitivity to and efficacy of drug therapy using tools like bioinformatics and pharmacogenomics. These fields, together with the evolving areas of genomics, proteomics, and metabolomics, constitute the premise and promise of personalized medicine.

Evidence-based medicine seeks to narrow the gap between clinical research and practice by explicitly and systematically focusing the attention of clinicians on the most up-to-date evidence from epidemiologic and clinical trial studies. Specifically, evidence-based medicine promotes the judicious use of meta-analyses of randomized controlled trials and other scientifically derived knowledge for clinical decision-making. However, an inherent weakness of the meta-analytical focus is that individuals vary greatly in regard to their manifestations of disease, symptoms, co-morbidities, genetic predisposition, and variance in molecular sensitivity to drugs, which cannot be reflected in guidelines derived from meta-analyses of the general patient population.

According to the US President’s Council of Advisors on Science and Technology,^10^

personalized medicine refers to the tailoring of medical treatment to the individual characteristics of each patient; […] the ability to classify individuals into subpopulations that differ in their susceptibility to a particular disease or their response to a specific treatment. Preventive or therapeutic interventions can then be concentrated on those who will benefit, sparing expense and side effects for those who will not.^10^

Given the large health and economic impact of DM, there is understandable interest in using personalized medicine strategies to identify those individuals who are most at risk of developing DM and its various complications, and who are most likely to benefit from a specific management strategy, in order to apply proven measures to delay or prevent their progression to DM and its subsequent complications.^11^,^12^ In this review we will provide an introduction to the principal personalized medicine tools and strategies, and provide examples of how they may be applied to diabetes, in particular to type 2 DM (DM2). This includes enhanced prediction of the onset and course of DM and its complications, treatment planning (choice of treatment modality), treatment prioritization and goal setting, and recognition of potential pathophysiologic mechanisms of DM and its complications.

## PERSONALIZED MEDICINE AND THE NEW “OMICS”

Taking advantage of high-throughput technological developments in the laboratory and advances in data management capabilities, it is now possible to acquire and analyze very large volumes of information from studies of genetic and metabolic markers from great numbers of individuals. This has led to the fields of genomics, proteomics, transcriptomics, metabolomics, and pharmacogenomics (see Glossary for definitions). Analyses of large numbers of variants from genome-wide association studies (GWAS), or of large numbers of protein and other metabolites in body fluids, from large cohorts that may number in the tens of thousands generate enormous amounts of data. The field of bioinformatics uses “big data” approaches to organize and usefully analyze these data sets to recognize patterns and associations that may have pathophysiologic, diagnostic, prognostic, or therapeutic utility.

These tools of personalized medicine may be used to predict risk for developing DM, as well as an individual patient’s risk of developing one or all of the complicating morbidities associated with DM, such as retinopathy, neuropathy, nephropathy, or large-vessel disease (macrovasculopathy). They also have potential to guide treatment planning, in terms of personalized goal setting, choice of treatments, and treatment prioritization.

### Genomics and Type 2 Diabetes Mellitus

Studies of the family medical history of those with DM2 as well as observation of differential incidence of DM in different ethnic groups have long pointed to a significant inherited component to DM2 susceptibility. Nevertheless, the rapid rise in DM2 incidence in the last few decades suggests the interaction of changes in environment and lifestyle with genetic predisposition. The principle of genome-wide association studies is to investigate differences in the prevalence of genetic variations (single nucleotide polymorphisms, SNPs) in DNA samples from populations with and without the condition of interest. Significant differences point to possible etiological associations with the condition. Recent expansion of genome-wide association studies to include “environment-wide associations” may help identify novel nutritional or other environmental interactions that modulate genetic predisposition to DM.^13^ After the successful cloning of the human genome, initial enthusiasm about the possibility of identifying the specific genetic basis for DM2 has been followed by the realization that a large number of genes contribute to DM2 susceptibility. These include CDKAL1, CDKN2A, and CDKN2B that influence β-cell mass; MTNR1B, TCF7L2, and KCNJ11 that influence β-cell function; FTO that is associated with obesity; and IRS1 and PPAR-γ that contribute to insulin resistance independent of obesity.^14^ Furthermore, there may be unique markers of genetic susceptibility to DM2 in certain ethnic groups with a high incidence of DM2,^15^,^16^ and interactions between individual genetic variants may also influence DM risk. For example, in a study of an Ashkenazi Jewish population, the presence of HNF4A or WFS1 SNPs was each associated with modestly increased risk of DM, while the presence of both increased that risk three-fold.^17^ Unfortunately, although genome-wide association studies have already identified over 65 gene variants related to DM2,^18^ predominantly involved in β-cell function,^19^ collectively they explain only a small portion (<10%) of DM2 heritability.^20^ Thus, while family history of DM approximately doubles the risk of developing DM, the genetic variants associated with DM risk have only a small effect on the ability to predict the future development of the disease.^21^ It is very likely that epigenetic changes contribute to familial clustering of risk for obesity and DM,^22^ changes that by definition are not detectable with genomic studies.

In contrast to DM2, a small number of monogenic defects have been recognized to cause the uncommon autosomal dominantly inherited forms of maturity-onset diabetes of the young (MODY).^23^ These defects disrupt β-cell function, and their recognition and precise genetic diagnosis is clinically important in directing treatment towards more effective and easier-to-use sulfonylurea drugs rather than insulin. The most common form (MODY3) results from a mutation of hepatocyte nuclear factor-1α on chromosome 12.^24^ In MODY2, a defective glucokinase gene on chromosome 7P results in disturbed β-cell sensing of glucose concentration.

### Transcriptomics and Type 2 Diabetes Mellitus

Sometimes referred to as gene expression profiling, transcriptomics is the quantitative study of all genes expressed in a given biological state^25^ and measures all of the various RNA forms (messenger, ribosomal, transfer, etc.) produced by DNA transcription in a particular cell or tissue. MicroRNAs are small, non-coding RNAs that are involved in control of gene expression and play an important role in regulating metabolic and cardiovascular processes.^26^ In combination with metabolomics, transcriptomic studies in animal models of DM have identified a number of novel genetic and metabolic changes, including differences in branched-chain amino acids, nicotinamide metabolites and pantothenic acid, that provide direction for additional studies of diabetes pathophysiology.^27^

### Proteomics and Type 2 Diabetes Mellitus

Techniques such as matrix-assisted laser desorption/ionization,^28^ mass spectroscopy, and electrospray ionization,^29^ alone or in combination, are used to identify and quantify all of the large number of protein products of a genome, in a specific tissue or body fluid. Differences associated with obesity, DM, or other disease states may identify novel pathogenic mechanisms, prognostic markers, or potential therapeutic targets. Study of proteomics is complementary to genomics, as post-translational modifications of proteins of potential importance to understanding the pathophysiology of DM and its complications in tissues such as adipose tissue or skeletal muscle will not be detected by genomic studies.^30^

### Metabolomics and Type 2 Diabetes Mellitus

Metabolomics uses tools such as nuclear magnetic resonance and mass spectroscopy to identify and quantitate large numbers of small-molecule products of metabolism. “Targeted” metabolomic studies are limited to a certain category of metabolites of interest (e.g. amino acids). In the field of DM, metabolomics has helped identify novel risk factors for DM, which may be useful biomarkers for early DM risk^31^,^32^ and may also serve as clues to increase understanding of the complex pathophysiology of DM2. Analysis of many metabolites in baseline samples from large prospective population studies, such as the Framingham Heart Study, has identified strong independent predictive relationships between levels of branched-chain and aromatic amino acids (isoleucine, leucine, valine, tyrosine, and phenylalanine) and risk of DM incidence over 12 years.^31^ Further studies in this population identified a novel metabolite (2-aminoadipic acid) which is independently predictive of DM risk, pointing to a potential different pathophysiologic pathway underlying DM.^33^ The field of “lipidomics” employs the analytic technology and large data set approach of metabolomics to study variations in lipid structures. Using the same Framingham Heart Study population, Rhee et al. found that shorter triacylglycerol fatty acid chain length and lower double-bond content reflect insulin resistance and serve as an independent marker of DM risk.^34^ The potential role of metabolomic studies in DM research and practice has recently been reviewed.^35^,^36^

### Pharmacogenomics and Type 2 Diabetes Mellitus

Pharmacogenomics studies the effect of genetic variations on drug kinetics or action. Genetically determined differences in absorption or metabolism of an agent, or variation in tissue responsiveness, may increase or decrease the effectiveness or side effects of a drug in a clinically important manner. Pharmacogenomic advances have the potential to improve the effectiveness and safety of oral anti-diabetic therapy^37^,^38^ but have not yet reached the stage of wide clinical applicability. This is in contrast to the field of antithrombotic therapy where variants in the CYP2C19 enzyme, which affect hepatic activation of the widely used anti-platelet agent clopidogrel, may result in clinically relevant reduction in drug effectiveness. Genetic testing for this variant is available, but its role in routine practice remains controversial.^39^ In the case of metformin, the most widely used drug for DM2, recent findings of the role of organic cationic transporter proteins in the mechanism of action of metformin led to the discovery that variants related to the genes for these transporter proteins may reduce metformin effectiveness^40^ and tolerance.^41^ A GWAS of DM2 patients linked responsiveness to metformin to a SNP associated with the gene for ATM (ataxia telangiectasia mutated).^42^ Although this genetic variant accounts for only 2.5% of the variation in metformin response, findings such as these facilitate understanding of drug mechanisms of action.

### Nutrigenetics and nutrigenomics

Nutrigenetics has been defined as the science of the effect of genetic variation on dietary response, while nutrigenomics studies the impact of nutrients and other elements of the diet on gene expression.^43^ These new fields recognize the major interactions between genetic make-up and response to diet and dietary changes, both in terms of predisposing to development of obesity, metabolic syndrome, and DM2, and in determining responsiveness to specific dietary changes. For example, while TCF7L2 (transcription factor 7–like 2 protein, which is involved in the synthesis, processing, and secretion of insulin) is strongly and consistently related to DM2 risk, this risk is modulated by dietary carbohydrate and is greater when the diet contains larger amounts of high glycemic-index foods.^44^

## PERSONALIZED MEDICINE AND PREDICTION OF DM2 RISK

The disordered metabolic state of type 2 DM is characterized by elevated levels of glucose, resulting from reduced effectiveness of insulin’s actions on its target tissues with an inadequate compensatory response of the insulin-secreting pancreatic islet β-cells.^45^ The precise glucose levels at which DM2 is diagnosed are necessarily arbitrary (based mainly on the threshold for presence of background retinopathy in epidemiological studies),^23^ such that many people who do not meet formal diagnostic criteria for DM2 nevertheless have abnormally elevated levels of glucose, along with a degree of insulin resistance and inadequate insulin secretion. Such individuals may already have evidence for diabetic complications and are at risk for progression of these abnormalities over time. A number of high-quality randomized controlled trials have demonstrated that risk of progression to DM can be cut in half,^46^ making it a priority to identify those at greatest risk who are candidates for primary prevention measures.^47^

Based on current American Diabetes Association recommendations,^23^ increased risk for DM2 (often termed “prediabetes”), may be identified in one of three ways: 1) fasting plasma glucose (FPG) of 100–125 mg/dL (characterized as impaired fasting glucose); 2) plasma glucose 2 hours after a 75-g oral glucose challenge of 140–199 mg/dL (impaired glucose tolerance); or 3) hemoglobin A1c (HbA1c) test of 5.7%–6.4%. These criteria do not identify identical groups of people at increased risk for DM2, and their pathophysiology and susceptibility to complications may differ. For example, those with impaired glucose tolerance are at greater risk for macrovascular complications, including stroke, than those with impaired fasting glucose.^48^,^49^

While the prevalence of prediabetes is now as high as 35% of US adults (50% of those 65 and older), only a small number of these (as few as 3%) develop DM2 each year.^50^ Even with the categorical diagnosis of prediabetes, an individual’s risk for progression to DM2 over 5 years can vary widely, from 100% (for those with HbA1c 6.0%–6.4% and FPG 116–125 mg/dL) to close to zero (for those with HbA1c < 6% and FPG < 110 mg/dL), based on prospective studies in a Japanese population.^51^ Thus a more precise personalized estimate of absolute risk for developing DM2 than is provided for by the broad categories of impaired fasting glucose, impaired glucose tolerance, and prediabetes is highly desirable.

Personalized medicine has the potential to improve prediction of DM2 risk. Simple clinical risk factors (age, weight, family history of DM) and simple laboratory measures (glucose, triglyceride) explain about 80% of the variance in DM incidence.^52^ Novel clinical/anthropometric risk factors for DM development continue to be reported.^53^ To date at least 65 genetic variants contributing to DM2 have been identified,^18^,^22^ but these account for less than 10% of cases. Initial studies with a limited number of DNA markers showed only modest incremental value of adding genetic data to clinical information in predicting risk for DM2,^21^,^54^,^55^ thus the potential for genomics to enhance prediction of DM2 risk remains unrealized.

While weight or body mass index (BMI) is consistently a strong determinant of metabolic syndrome and DM2, individuals with the same weight or BMI may have very different risks of DM2. A personalized assessment of the metabolic impact of obesity needs to take into account the distribution pattern of the excessive adipose tissue. Intra-abdominal visceral and in particular hepatic fat accumulation is associated with insulin resistance and systemic inflammation, with increased risk for metabolic syndrome, DM2, and cardiovascular disease, while excess subcutaneous fat does not impair insulin sensitivity, leading to the concept of metabolically “benign versus malign” obesity.^56^

A large number of additional novel risk factors (including FEV1, adiponectin, leptin, gamma-glutamyltransferase, ferritin, inter-cellular adhesion molecule 1, complement C3, white blood cell count, albumin, activated partial thromboplastin time, coagulation factor VIII, magnesium, hip circumference, and heart rate) are each independently associated with risk for DM2 but add little or nothing to basic clinical prediction models in predicting incident DM2.^57^ Sex hormone-binding globulin (SHBG), traditionally considered to be a passive transporter protein for sex steroids, may have a more active role in DM causation. Observational studies identified lower levels of SHBG as a risk factor for insulin resistance and incident DM, and *in-vitro* studies demonstrated G-protein-linked receptor-mediated effects of SHBG on intracellular processes related to insulin resistance.^58^ Multiple confounding factors (e.g. obesity, hyperinsulinemia) are associated with lower SHBG and risk for DM2; however, recent genetic studies suggest an independent role for sex steroids and SHBG in the etiology of DM2.^59^

In recent years, metabolomic studies of large numbers of metabolites in blood and/or urine have identified novel predictors of DM risk, e.g. circulating levels of aromatic and branch-chained amino acids, which are independent predictors of insulin resistance^60^ and DM risk. Metabolomic studies have identified novel pathophysiological mediators of metabolic syndrome, such as nicotinuric acid.^61^ Using a targeted metabolomic approach and measuring over 160 serum metabolites with flow injection analysis tandem mass spectrometry in prospectively collected samples from large population-based studies, Floegel et al. identified a number of changes in sugar metabolites, amino acids, and choline-containing phospholipids that modestly improve prediction of DM risk.^62^ Identifying such metabolomic markers may prove to be useful in directing studies of the associated genes in at-risk populations.^63^

## PREDICTING TYPE 1 DM RISK

Autoimmune-mediated destruction of the insulin producing β-cells of the pancreatic islets results in type 1 DM. Increased risk for developing type 1 DM may be recognized by a family history of type 1 DM or other autoimmune diseases, by the presence in the blood of a range of antibodies to insulin and islet-related antigens (e.g. islet-cell antibodies, insulin autoantibodies, antibodies to glutamic acid decarboxylase), or by the identification of a “high-risk” HLA type.^64^ Recently genomic studies combined with bioinformatics techniques have been able to identify a small number of SNPs that can rapidly and inexpensively predict the presence of the high-risk HLA-DR/DQ types,^64^ which may facilitate identification of those individuals who are candidates for studies of interventions to prevent complete β-cell loss and thereby prevent or ameliorate the type 1 DM.^65^

## PERSONALIZED MEDICINE AND CHRONIC MICROVASCULAR COMPLICATIONS OF DM

As a function of time and extent of hyperglycemic burden, individuals with DM are prone to develop renal, retinal, or neurological damage that can result in renal failure, blindness, disabling pain, or lower-extremity amputations. However, not all patients with DM develop these complications, regardless of duration or degree of hyperglycemic control. Fifteen to twenty years after diagnosis of DM, 50%–80% have evidence for retinopathy,^66^ only a minority of which is vision-threatening, up to 30% have increased levels of albumin in the urine (an early stage in the development of nephropathy),^67^ and about 50% have symptoms of peripheral neuropathy.^68^ Randomized controlled trials, including DCCT,^69^ UPKDS,^70^ Kumamoto,^71^ ACCORD,^72^ and ADVANCE,^73^ demonstrate the potential to reduce or delay some or all of these risks by controlling hyperglycemia. It has also become apparent that uncontrolled hyperglycemia early in the course of DM may result in sustained increased risk of complication development, regardless of subsequent glycemic control. This concept of “metabolic memory” may reflect epigenetic changes (e.g. DNA methylation and post-translational histone modification).^74^ Personalized management of complication risk would be greatly enhanced by improved discrimination of those not destined to develop the complication from those who would most benefit from aggressive measures to reduce their risk.

### Diabetic Nephropathy Prediction and Prevention

Nephropathy occurring as a complication of type 1 and type 2 DM is characterized clinically by increased levels of protein in the urine, declining glomerular filtration rate, hypertension, and eventual progression to renal failure, requiring renal replacement therapy with dialysis or transplantation. Not all patients with DM develop albuminuria, and this is not always progressive. Progression may be slowed by excellent glycemic and blood pressure control, as well as use of angiotensin-converting enzyme inhibitor medications.^75^

Numerous clinical factors are associated with risk for nephropathy (blood pressure, age, obesity, extent of hyperglycemia). There is also a clear inherited (familial and racial) contribution to nephropathy susceptibility. Although genome-wide association studies have not identified definite DM nephropathy susceptibility loci in DM2, ongoing family studies may provide clues to uncommon gene variants that increase nephropathy risk.^76^ Studies to date have also not clearly confirmed a specific gene marker associated with nephropathy in type 1 DM.^77^ Transcriptomic studies of non-coding RNA molecules involved in regulation of gene expression point to their role in influencing renal response to hyperglycemia,^78^ and measurement of specific microRNAs in the urine may improve prediction of risk for development and progression of DM nephropathy.^78^ New proteomic techniques may permit earlier recognition, and therefore more directed treatment, of those at risk for DM nephropathy.^79^ One such novel urinary marker is liver-type fatty acid-binding protein, which may enhance prediction of risk for progression of early nephropathy in type 1 DM.^80^ The ability to identify diabetic patients not at risk for future nephropathy would permit relaxed screening and treatment recommendations.

### Diabetic Retinopathy Prediction and Prevention

Eye changes in DM result from abnormal retinal microvasculature (microaneurysms with abnormal permeability as well as vascular occlusion with consequent ischemia and neovascularization).^81^ Background retinopathy changes may be evident at the time of diagnosis of DM2 and eventually develop in the majority of type 1 and type 2 DM patients. Only a minority of these progress to vision-threatening proliferative retinopathy, typically as a function of time and degree of glycemic control, especially in the presence of other complications like nephropathy or non-healing foot ulcers.^82^ Medical interventions are effective in arresting the progresssion of vision-threatening retinopathy, forming the basis of current screening recommendations for asymptomatic retinopathy in all type 1 and type 2 DM.^4^–^6^ Along with improved glycemic control in recent decades, this has led to a declining incidence and severity of diabetic retinopathy in the USA.^83^ In recent years genomic studies have identified potential genetic associations with DM retinopathy risk, for example the gene encoding the receptor for advanced glycation end products (RAGE, especially the 1704T allele)^84^ and the gene for methylenetetrahydrofolate reductase (MTHFR),^85^ where the 677C/T polymorphism has been associated with modestly increased risks for nephropathy and retinopathy. Investigators have recently reported use of proteomic methods to study proteins in the aqueous humor of the eye that may provide insights into the pathophysiology of DR,^86^ but proteomic and genomic testing for diabetic retinopathy risk are not yet useful in clinical practice.

### Diabetic Neuropathy Prediction and Prevention

Peripheral nerve dysfunction results from metabolic as well as microvascular damage and may lead to significant pain, as well as loss of sensation predisposing to lower-extremity amputation. Autonomic neuropathies affect gastrointestinal motility and can lead to cardiac dysfunction. Risk for neuropathy rises with duration of DM, degree of hypertension and hyperglycemia, as well as smoking.^87^ Vitamin D insufficiency may also be an independent predictor of developing neuropathy symptoms.^68^ Nevertheless, about 50% of DM patients appear resistant to these factors and do not develop neuropathy. Recent proteomic studies of patients with diabetic neuropathy have identified a number of proteins, including a fragment of the apolipoprotein C-I precursor, that associate with diabetic neuropathy.^88^ Metabolomic studies have identified phospholipid biomarkers that may improve discrimination between those DM patients with and without neuropathy.^89^ Such advances may lead to improved assessment of neuropathy risk and may enhance understanding of the pathophysiology of diabetic neuropathy.

## PERSONALIZED MEDICINE AND CHRONIC MACROVASCULAR COMPLICATIONS OF DM

While historically much attention was focused on preventing the aforementioned microvascular complications of DM, in reality the most significant area of preventable DM-related morbidity, mortality, and heath care utilization^90^ is arteriosclerotic narrowing in the coronary, cerebrovascular, and peripheral arterial beds. This results in the devastating manifestations of angina pectoris, acute myocardial infarction, sudden cardiac death, heart failure, stroke, intermittent claudication, and lower-extremity amputation. Risk of atherosclerotic cardiovascular disease (ASCVD) rises with fasting glucose even in the “prediabetes” range.^91^ While glycemic control in the short or medium term appears to have little impact on the course of these macrovascular manifestations, long-term follow-up studies from the DCCT^92^ and the UKPDS^70^ trials (including type 1 and type 2 DM patients, respectively) showed a beneficial long-term “legacy” effect of early glycemic control on long-term macrovascular disease risk. In contrast to glycemic control, there is strong evidence that addressing other cardiac risk factors (encouraging smoking cessation, use of angiotensin-converting enzyme inhibitor drugs, control of blood pressure and elevated LDL-cholesterol, as well as use of anti-platelet agents) substantially lowers short- and long-term risk of macrovascular events in those with DM2.^93^ A clinically important barrier to therapy with HMG-CoA reductase inhibitors (“statins”) in DM is the occurrence of muscular symptoms, which typically are mild (aching, weakness) but rarely may be severe or life-threatening (rhabdomyolysis). Recent pharmacogenetic studies found that variants in the SLCO1B1 gene (affecting cytochrome-mediated drug clearance) are associated with an increased risk of statin-induced myopathy,^94^ particularly with simvastatin but not pravastatin.

In some studies, those with DM2 but without history of cardiac events bear the same risk of experiencing a cardiac event as non-DM patients who have already experienced an event.^95^ As a result, primary prevention of ASCVD in DM2 is treated in the same way as secondary prevention in those without DM (“DM as a coronary disease equivalent”).^4^ Consequently, patients with DM2 typically are exposed to the costs, complexity, and risk of side effects from poly-pharmacy, receiving multiple medications to lower LDL-cholesterol and blood pressure as well as glucose. Improved assessment of ASCVD risk would allow for a more personalized implementation of these preventive measures. More than a dozen models have been developed to predict absolute risk for ASCVD in DM2 patients, which vary in their predictive power (AUC ranging from 0.61 to 0.86), validation, and evidence for impact on clinical practice and outcomes.^96^ Estimates of ASCVD risk need to take into account ethnicity.^97^ All use clinical variables (such as age, gender, HbA1c, duration of DM, presence of albuminuria, tobacco use, measures of blood pressure, and lipid parameters). None incorporate novel risk factors such as soluble receptors for advanced glycation end products (sRAGE),^98^ hsCRP or other measures of inflammation, markers of endothelial dysfunction, or growth factors such as placental growth factor or transforming growth factor-β that are associated with increased cardiac risk.^99^ None to date include genomic, proteomic, or metabolomic information.

A novel predictor of ASCVD risk in those with both type 1 and type 2 DM is the haptoglobin genotype.^100^ Haptoglobin is a circulating hemoglobin-scavenging protein that exists in three variants: 1–1, 1–2, and 2–2. A number of studies identified a doubled risk for ASCVD for those with the 2–2 genotype,^100^ which is present in approximately 36% of DM2. An intriguing pharmacogenomic finding from intervention trials is that only those DM patients with the 2–2 genotype appear to respond to anti-oxidant treatment (vitamin E) with reduced ASCVD risk of up to 50%.^100^ If these findings are confirmed, then testing for haptoglobin genotype of all DM patients could be recommended, with addition of vitamin E treatment to reduce ASCVD risk for those with the 2–2 genotype.

Genomic approaches (GWAS) not specifically in patients with DM have identified more than 20 variants (SNPs) that are associated with increased risk for coronary artery disease.^101^ In patients with DM2, a genetic predisposition score derived from GWAS of DM2 predisposition was independently associated with risk for cardiovascular complications,^102^ pointing to an overlapping etiological basis for DM2 and ASCVD. However, it is not clear that genomic information enhances the more traditional clinical risk factor approach to ASCVD prediction.^103^ Nevertheless, genomic studies of coronary artery disease, as with DM2 itself, have potential to improve understanding of pathophysiology, predicttion, prognosis, diagnosis, and management.^104^

Studies of circulating microRNA in patients with DM found that presence of peripheral vascular complications in DM is associated with loss of endothelial mIR-126, possibly due to disturbed fibrinolysis.^26^ This field of study has potential to increase understanding of the pathophysiology of diabetic macrovasculopathy. Proteomic studies of vascular tissue, plaque, and body fluids from patients with atherosclerosis have been performed, with some progress in identifying potential biomarkers of disease activity or disease risk, as well as proteins of potential pathophysiological significance. Proteomic approaches have identified unusual apolipoprotein patterns in the small dense LDL of insulin-resistant patients with DM and metabolic syndrome that may help explain their susceptibility to ASCVD.^105^

## PERSONALIZED MEDICINE AND DM TREATMENT

A goal as yet unrealized in the clinical management of patients with DM is to use genomic, metabolic, and other data to predict which patients will progress to a particular complication of DM, in order to establish an indication for specific preventive interventions. Within the realm of preventive therapy, the ideal situation would be the ability to predict individual responsiveness to and tolerance of a particular treatment, in order to design the most effective and best-tolerated individual program of drug, dietary, and exercise therapies. There has been modest progress in understanding the pharmacogenomics of the glucose-lowering medications,^37^ but practical implementation remains elusive. Thus, choice amongst drugs and drug classes for DM remains largely empirical.^8^

Compared to the field of pharmacogenomics there has been less research into the genetic determinants of responsiveness to dietary change or increased physical activity, two key modalities in the prevention and treatment of DM. Intriguing recent studies point to differential sensitivity to particular dietary regimens based on genotype. Genome-wide association studies have identified a number of genetic loci that associate with BMI,^106^ one of the most consistent of which is the “fat mass and obesity associated gene” (FTO). Interaction between FTO variants and diet and exercise has been found. The interaction between FTO and risk of obesity is modulated by exercise, in that increased levels of physical activity attenuate the rise in weight seen in men carrying the FTO rs1861868 SNP.^107^ Interaction with diet has also been found, with recent randomized trial data suggesting that individuals with the FTO variant rs1558902 showed enhanced changes in weight, body composition, and superficial fat mass in response to a high-protein diet,^108^ while subjects with the TCF7L2 rs12255372 genotype showed greater reduction in weight and DM risk by consumption of a low fat (20%) diet.^109^ If these kinds of findings are confirmed, specific dietary prescription for patients with obesity and DM2 may be aided by genomic testing. However, it is not clear that information about genetic risk influences behavior in a clinically useful manner. A recent randomized control trial found no significant effect of counseling on personalized genetic risk for DM2 on participation in and outcome of a lifestyle change program to prevent DM2.^110^

A recent GWAS linked genetic variants in the SGIP1, CYP19A1, and LEPR genes to voluntary leisure-time activity, independently of BMI. Even though these effects were small, studies such as this point to possible explanations for variations in habitual exercise activity and related health consequences.^111^ Great variation in individual responses to exercise training has long been recognized, both in terms of improved muscle strength and aerobic performance. Genetic determinants underlying this variation have been uncovered. Variants in the ISIG2 gene (a gene associated with obesity) contribute to variation in subcutaneous fat in women and to attenuation of the effects of resistance training in men.^112^ Variants in the genes for CCL2 (chemokine (C-C motif) ligand 2) and its receptor (CCR2), a chemokine related to muscle repair and response to exercise, influence muscle strength and response to strength training.^113^ In spite of these preliminary findings, “exercise prescription” for patients with DM remains largely empirical, and clearly much research remains to be done in order to understand adequately the individual variation in response to physical training,^114^ and in order to match optimally the exercise recommendations to individual patients with DM.

## ECO-SYSTEM IN PERSONALIZED MEDICINE

Improved diet and exercise are hallmarks of DM prevention and treatment. However, they are difficult to sustain. When prescribing such treatments, the caregiver has to be aware of the patient’s eco-system at the point of care. For example, a project involving a US Veterans Administration’s data set has been recently launched in order to apply personalized medicine at the point of care. This data set contains 10 million patient records with demographic, clinical, and genomic data. The demographic data will be analyzed and processed to render approximate geolocation. A high-performance query interface will be enabled to co-query records based on geography, clinical, and genomic attributes. Interactive data maps and heat maps will be created. The data set will be mined for the derivation of knowledge, and, utilizing The Terra Fly Geospatial Analytics System (http://terrafly.com), correlates of eco-system components with DM and obesity will be determined. For example, studies have indicated that residents of neighborhoods without sidewalks tend to be overweight.^115^ The absence of sidewalks seems to be a factor in discouraging people from walking, thus reducing the potential benefits of this simple exercise to prevent and treat DM. The presence of sidewalks is automatically derivable from analysis of aerial and satellite images and property boundaries represented by polygons; it allows correlation of findings from imagery analysis and the obesity demographics statistics.

## PERSONALIZED MEDICINE AND DM TREATMENT TARGETS

Recent guidelines recommend moving away from uniform glycemic control goals for people with DM,^4^,^8^ with the result that the majority of DM patients may not be candidates for the most aggressive HbA1c goals.^116^ Personalization of glycemic control target is based on clinical parameters, including age, duration of DM, and presence of DM complications or co-morbidities, and eco-system components. If microvascular or macrovascular risk could be more precisely assessed than currently, more or less aggressive treatment targets could be used, not just for glucose, but also for blood pressure and lipid lowering treatments.

## CONCLUSIONS

Patients, physicians, health care organizations, and policy planners are grappling with the worldwide rise in incidence of DM. Diabetes mellitus and its related complications cause substantial morbidity and mortality and are consuming an increasing proportion of health care budgets. There is wide individual and ethnic variation in susceptibility to DM as well as environmental factors, making a “one size fits all” approach to DM management inefficient. The vision of DM care in the era of personalized medicine is that patients and physicians, using decision support systems embedded in the electronic medical record at the point of care, will have access to the results of individualized genomic, proteomic, and metabolic information, as well as the most current evidence-based guidelines and literature updates.^12^ This will provide them with up-to-date, accurate, and actionable information on risk for DM and its diverse manifestations, allowing them jointly to prioritize and optimize diagnostic, treatment, and monitoring plans. In this way, the most cost-effective and best-tolerated treatments can be directed at the manifestations of disease most likely to impact that individual’s health and life expectancy, while avoiding treatments that are unlikely to be of benefit. The tools of personalized medicine have made substantial progress towards understanding the pathophysiologic mechanisms behind individual variation in DM and its manifestations.

## Abbreviations:

ASCVD: atherosclerotic cardiovascular disease;
BMI: body mass index;
DM: diabetes mellitus;
DM2: type 2 diabetes mellitus;
GWAS: genome-wide association study;
HbA1c: hemoglobin A1c;
MODY: maturity-onset diabetes of the young;
SNP: single nucleotide polymorphism.

## GLOSSARY

**Table:**

**Term**	**Definition**	**Examples of Techniques Used**
Personalized medicine	“The tailoring of medical treatment to the individual characteristics of each patient”^10^	
Genomics	“The study of all of a person’s genes (the genome), including interactions of those genes with each other and with the person’s environment”^117^	Genome-wide association studies
Genome-wide association study (GWAS)	“An approach used in genetics research to look for associations between many (typically hundreds of thousands) specific genetic variations (most commonly single-nucleotide polymorphisms) and particular diseases”^118^	
Epigenetics	“Changes in gene expression and cellular phenotypes that are mitotically stable but that occur without accompanying changes in primary DNA sequence”^22^	Studies of gene methylation patterns
Transcriptomics	“The quantitative study of all genes expressed in a given biological state”^25^	Gene expression microarrays; RNA sequencing^25^
Proteomics	Large-scale analysis of all the proteins in an organism, tissue type, or cell (called the proteome). Proteomics can be used to reveal specific, abnormal proteins that lead to diseases	Matrix-assisted laser desorption/ionization^28^; mass spectroscopy; electrospray ionization^29^
Metabolomics (metabolic profiling)	“Measurements of the metabolome, which represents the entire collection of all small-molecule metabolites present in any biological organism”^36^	Nuclear magnetic resonance; mass spectrometry^36^
Pharmacogenomics	“Pharmacogenomics is the study of an individual’s interaction with a specific drug based upon the genetic make-up of the individual”^39^	“Pharmacogenomics studies the influence of genetic variations on the patient’s response to specific drugs, such as the correlation between the efficacy or toxicity of a certain drug and a specific gene expression or a single-nucleotide polymorphism”^39^
Bioinformatics	“Information technology as applied to the life sciences, especially the technology used for the collection and analysis of genomic data”^118^	

## References

[1] Whiting DR, Guariguata L, Weil C, Shaw J (2011). IDF diabetes atlas: global estimates of the prevalence of diabetes for 2011 and 2030. Diabetes Res Clin Pract.

[2] Miller RG, Secrest AM, Sharma RK, Songer TJ, Orchard TJ (2012). Improvements in the life expectancy of type 1 diabetes: the Pittsburgh Epidemiology of Diabetes Complications study cohort. Diabetes.

[3] Gaede P, Lund-Andersen H, Parving HH, Pedersen O (2008). Effect of a multifactorial intervention on mortality in type 2 diabetes. N Engl J Med.

[4] (2013). American Diabetes Association. Standards of medical care in diabetes--2013. Diabetes Care.

[5] International Diabetes Federation 2012 Clinical Guidelines Task Force. Global Guideline for Type 2 Diabetes. http://www.idf.org/global-guideline-type-2-diabetes-2012.

[6] National Collaborating Centre for Chronic Conditions Type 2 Diabetes National Clinical Guideline for Management in Primary and Secondary Care (update).

[7] Stark Casagrande S, Fradkin JE, Saydah SH, Rust KF, Cowie CC (2013). The prevalence of meeting A1C, blood pressure, and LDL goals among people with diabetes, 1988–2010. Diabetes Care.

[8] Inzucchi SE, Bergenstal RM, Buse JB (2012). Management of hyperglycemia in type 2 diabetes: a patient-centered approach: position statement of the American Diabetes Association (ADA) and the European Association for the Study of Diabetes (EASD). Diabetes Care.

[9] Sherwin R, Jastreboff AM (2012). Year in diabetes 2012: the diabetes tsunami. J Clin Endocrinol Metab.

[10] President’s Council of Advisors on Science and Technology (2008). Priorities for Personalized Medicine.

[11] Spiegel AM, Hawkins M (2012). ‘Personalized medicine’ to identify genetic risks for type 2 diabetes and focus prevention: can it fulfill its promise?. Health Aff (Millwood).

[12] Zolotov S, Ben Yosef D, Rishe ND, Yesha Y, Karnieli E (2011). Metabolic profiling in personalized medicine: bridging the gap between knowledge and clinical practice in Type 2 diabetes. Pers Med.

[13] Patel CJ, Chen R, Kodama K, Ioannidis JP, Butte AJ (2013). Systematic identification of interaction effects between genome- and environment-wide associations in type 2 diabetes mellitus. Hum Genet.

[14] McCarthy MI (2010). Genomics, type 2 diabetes, and obesity. N Engl J Med.

[15] Saxena R, Saleheen D, Been LF (2013). Genome-wide association study identifies a novel locus contributing to type 2 diabetes susceptibility in Sikhs of Punjabi origin from India. Diabetes.

[16] Zhang R, Jiang F, Hu C (2013). Genetic variants of LPIN1 indicate an association with Type 2 diabetes mellitus in a Chinese population. Diabet Med.

[17] Neuman RJ, Wasson J, Atzmon G (2010). Gene-gene interactions lead to higher risk for development of type 2 diabetes in an Ashkenazi Jewish population. PLoS One.

[18] Sanghera DK, Blackett PR (2012). Type 2 Diabetes genetics: beyond. GWAS J Diabetes Metab.

[19] Dupuis J, Langenberg C, Prokopenko I (2010). New genetic loci implicated in fasting glucose homeostasis and their impact on type 2 diabetes risk. Nat Genet.

[20] Herder C, Roden M (2011). Genetics of type 2 diabetes: pathophysiologic and clinical relevance. Eur J Clin Invest.

[21] Lyssenko V, Jonsson A, Almgren P (2008). Clinical risk factors, DNA variants, and the development of type 2 diabetes. N Engl J Med.

[22] Drong AW, Lindgren CM, McCarthy MI (2012). The genetic and epigenetic basis of type 2 diabetes and obesity. Clin Pharmacol Ther.

[23] (2013). American Diabetes Association. Diagnosis and classification of diabetes mellitus. Diabetes Care.

[24] Thanabalasingham G, Pal A, Selwood MP (2012). Systematic assessment of etiology in adults with a clinical diagnosis of young-onset type 2 diabetes is a successful strategy for identifying maturity-onset diabetes of the young. Diabetes Care.

[25] Pedrotty DM, Morley MP, Cappola TP (2012). Transcriptomic biomarkers of cardiovascular disease. Prog Cardiovasc Dis.

[26] Zampetaki A, Mayr M (2012). MicroRNAs in vascular and metabolic disease. Circ Res.

[27] Connor SC, Hansen MK, Corner A, Smith RF, Ryan TE (2010). Integration of metabolomics and transcriptomics data to aid biomarker discovery in type 2 diabetes. Mol Biosyst.

[28] Insenser M, Montes-Nieto R, Vilarrasa N (2012). A nontargeted proteomic approach to the study of visceral and subcutaneous adipose tissue in human obesity. Mol Cell Endocrinol.

[29] Ramachandran S, Venugopal A, Sathisha K (2012). Proteomic profiling of high glucose primed monocytes identifies cyclophilin A as a potential secretory marker of inflammation in type 2 diabetes. Proteomics.

[30] Ohlendieck K (2012). Pathobiochemical changes in diabetic skeletal muscle as revealed by mass-spectrometry-based proteomics. J Nutr Metab.

[31] Wang TJ, Larson MG, Vasan RS (2011). Metabolite profiles and the risk of developing diabetes. Nat Med.

[32] Würtz P, Tiainen M, Mäkinen VP (2012). Circulating metabolite predictors of glycemia in middle-aged men and women. Diabetes Care.

[33] Wang TJ, Ngo D (2013). Psychogios N, et al. 2-Aminoadipic acid is a biomarker for diabetes risk. J Clin Invest.

[34] Rhee EP, Cheng S, Larson MG (2011). Lipid profiling identifies a triacylglycerol signature of insulin resistance and improves diabetes prediction in humans. J Clin Invest.

[35] McKillop AM, Flatt PR (2011). Emerging applications of metabolomic and genomic profiling in diabetic clinical medicine. Diabetes Care.

[36] Friedrich N (2012). Metabolomics in diabetes research. J Endocrinol.

[37] Manolopoulos VG, Ragia G, Tavridou A (2011). Pharmacogenomics of oral antidiabetic medications: current data and pharmacoepigenomic perspective. Pharmacogenomics.

[38] Bozkurt O, de Boer A, Grobbee DE, Heerdink ER, Burger H, Klungel OH (2007). Pharmacogenetics of glucose-lowering drug treatment: a systematic review. Mol Diagn Ther.

[39] Holmes DR (2013). Pharmacogenomic testing and antithrombotic therapy: ready for prime time?. Rambam Maimonides Med J.

[40] Viollet B, Guigas B, Sanz Garcia N, Leclerc J, Foretz M, Andreelli F (2012). Cellular and molecular mechanisms of metformin: an overview. Clin Sci (Lond).

[41] Tarasova L, Kalnina I, Geldnere K (2012). Association of genetic variation in the organic cation transporters OCT1, OCT2 and multidrug and toxin extrusion 1 transporter protein genes with the gastrointestinal side effects and lower BMI in metformin-treated type 2 diabetes patients. Pharmacogenet Genomics.

[42] Zhou K, Bellenguez C, Spencer CCA (2011). Common variants near ATM are associated with glycemic response to metformin in type 2 diabetes. Nat Genet.

[43] Fenech M, El-Sohemy A, Cahill L (2011). Nutrigenetics and nutrigenomics: viewpoints on the current status and applications in nutrition research and practice. J Nutrigenet Nutrigenomics.

[44] Cornelis MC, Qi L, Kraft P, Hu FB (2009). TCF7L2, dietary carbohydrate, and risk of type 2 diabetes in US women. Am J Clin Nutr.

[45] DeFronzo RA (2009). Banting Lecture. From the triumvirate to the ominous octet: a new paradigm for the treatment of type 2 diabetes mellitus. Diabetes.

[46] Gillies CL, Abrams KR, Lambert PC (2007). Pharmacological and lifestyle interventions to prevent or delay type 2 diabetes in people with impaired glucose tolerance: systematic review and meta-analysis. BMJ.

[47] Glauber HS, Karnieli E (2013). Preventing type 2 diabetes mellitus. A call for personalized intervention. The Permanente Medical Journal.

[48] DECODE Study Group, the European Diabetes Epidemiology Group (2001). Glucose tolerance and cardiovascular mortality: comparison of fasting and 2-hour diagnostic criteria. Arch Intern Med.

[49] Lee M, Saver JL, Hong KS, Song S, Chang KH, Ovbiagele B (2012). Effect of pre-diabetes on future risk of stroke: meta-analysis. BMJ.

[50] Centers for Disease Control and Prevention (2011). National diabetes fact sheet, 2011.

[51] Heianza Y, Arase Y, Fujihara K (2012). Screening for pre-diabetes to predict future diabetes using various cut-off points for HbA1c and impaired fasting glucose: the Toranomon Hospital Health Management Center Study 4. Diabet Med.

[52] Abbasi A, Peelen LM, Corpeleijn E (2012). Prediction models for risk of developing type 2 diabetes: systematic literature search and independent external validation study. BMJ.

[53] Jahangiri Noudeh Y, Hadaegh F, Vatankhah N (2013). Wrist circumference as a novel predictor of diabetes and prediabetes: results of cross-sectional and 8.8-year follow-up studies. J Clin Endocrinol Metab.

[54] Meigs JB, Shrader P, Sullivan LM (2008). Genotype score in addition to common risk factors for prediction of type 2 diabetes. N Engl J Med.

[55] Cornelis MC, Qi L, Zhang C, Kraft P (2009). Joint effects of common genetic variants on the risk for type 2 diabetes in U.S. men and women of European ancestry. Ann Intern Med.

[56] Kantartzis K, Machann J, Schick F (2011). Effects of a lifestyle intervention in metabolically benign and malign obesity. Diabetologia.

[57] Raynor LA, Pankow JS, Duncan BB (2013). Novel risk factors and the prediction of type 2 diabetes in the Atherosclerosis Risk in Communities (ARIC) study. Diabetes Care.

[58] Wallace IR, McKinley MC, Bell PM, Hunter SJ (2013). Sex hormone binding globulin and insulin resistance. Clin Endocrinol (Oxf).

[59] Perry JR, Weedon MN, Langenberg C (2010). Genetic evidence that raised sex hormone binding globulin (SHBG) levels reduce the risk of type 2 diabetes. Hum Mol Genet.

[60] Wurtz P, Soininen P, Kangas AJ (2013). Branched-chain and aromatic amino acids are predictors of insulin resistance in young adults. Diabetes Care.

[61] Huang CF, Cheng ML, Fan CM, Hong CY, Shiao MS (2013). Nicotinuric acid: a potential marker of metabolic syndrome through a metabolomics-based approach. Diabetes Care.

[62] Floegel A, Stefan N, Yu Z (2013). Identification of serum metabolites associated with risk of type 2 diabetes using a targeted metabolomic approach. Diabetes.

[63] Xie W, Wood AR, Lyssenko V (2013). Genetic variants associated with glycine metabolism and their role in insulin sensitivity and type 2 diabetes. Diabetes.

[64] Nguyen C, Morahan G, Varney M, Harrison L (2013). Definition of high risk type 1 diabetes HLA-DR and HLA-DQ types using only three single nucleotide polymorphisms. Diabetes.

[65] Staeva TP, Chatenoud L, Insel R, Atkinson MA (2013). Recent lessons learned from prevention and recent-onset type 1 diabetes immunotherapy trials. Diabetes.

[66] Klein R, Klein BE, Moss SE, Davis MD, DeMets DL (1984). The Wisconsin epidemiologic study of diabetic retinopathy. III. Prevalence and risk of diabetic retinopathy when age at diagnosis is 30 or more years. Arch Ophthalmol.

[67] Hovind P, Tarnow L, Rossing P (2004). Predictors for the development of microalbuminuria and macroalbuminuria in patients with type 1 diabetes: inception cohort study. BMJ.

[68] Soderstrom LH, Johnson SP, Diaz VA, Mainous AG (2012). Association between vitamin D and diabetic neuropathy in a nationally representative sample: results from 2001–2004 NHANES. Diabet Med.

[69] The Diabetes Control and Complications Trial Research Group (1993). The effect of intensive treatment of diabetes on the development and progression of long-term complications in insulin-dependent diabetes mellitus. N Engl J Med.

[70] Holman RR, Paul SK, Bethel MA, Matthews DR, Neil HA (2008). 10-year follow-up of intensive glucose control in type 2 diabetes. N Engl J Med.

[71] Ohkubo Y, Kishikawa H, Araki E (1995). Intensive insulin therapy prevents the progression of diabetic microvascular complications in Japanese patients with non-insulin-dependent diabetes mellitus: a randomized prospective 6-year study. Diabetes Res Clin Pract.

[72] Ismail-Beigi F, Craven T, Banerji MA (2010). Effect of intensive treatment of hyperglycaemia on microvascular outcomes in type 2 diabetes: an analysis of the ACCORD randomised trial. Lancet.

[73] Patel A, MacMahon S, Chalmers J, ADVANCE Collaborative Group (2008). Intensive blood glucose control and vascular outcomes in patients with type 2 diabetes. N Engl J Med.

[74] Keating ST, El-Osta A (2013). Epigenetic changes in diabetes. Clin Genet.

[75] Coca SG, Ismail-Beigi F, Haq N, Krumholz HM, Parikh CR (2012). Role of intensive glucose control in development of renal end points in type 2 diabetes mellitus: systematic review and meta-analysis intensive glucose control in type 2 diabetes. Arch Intern Med.

[76] Pezzolesi MG, Krolewski AS (2013). The genetic risk of kidney disease in type 2 diabetes. Med Clin North Am.

[77] Williams WW, Salem RM, McKnight AJ (2012). Association testing of previously reported variants in a large case-control meta-analysis of diabetic nephropathy. Diabetes.

[78] Alvarez ML, Distefano JK (2013). The role of non-coding RNAs in diabetic nephropathy: potential applications as biomarkers for disease development and progression. Diabetes Res Clin Pract.

[79] Zürbig P, Jerums G, Hovind P (2012). Urinary proteomics for early diagnosis in diabetic nephropathy. Diabetes.

[80] Panduru NM, Forsblom C, Saraheimo M (2013). Urinary liver-type fatty acid-binding protein and progression of diabetic nephropathy in type 1 diabetes. Diabetes Care.

[81] Antonetti DA, Klein R, Gardner TW (2012). Diabetic retinopathy. N Engl J Med.

[82] Nwanyanwu KH, Talwar N, Gardner TW, Wrobel JS, Herman WH, Stein JD (2013). Predicting development of proliferative diabetic retinopathy. Diabetes Care.

[83] Lecaire T, Palta M, Zhang H, Allen C, Klein R, D’Alessio D (2006). Lower-than-expected prevalence and severity of retinopathy in an incident cohort followed during the first 4-14 years of type 1 diabetes: the Wisconsin Diabetes Registry Study. Am J Epidemiol.

[84] Niu W, Qi Y, Wu Z, Liu Y, Zhu D, Jin W (2012). A meta-analysis of receptor for advanced glycation end products gene: four well-evaluated polymorphisms with diabetes mellitus. Mol Cell Endocrinol.

[85] Niu W, Qi Y (2012). An updated meta-analysis of methylenetetrahydrofolate reductase gene 677C/T polymorphism with diabetic nephropathy and diabetic retinopathy. Diabetes Res Clin Pract.

[86] Chiang SY, Tsai ML, Wang CY (2012). Proteomic analysis and identification of aqueous humor proteins with a pathophysiological role in diabetic retinopathy. J Proteomics.

[87] Tesfaye S, Selvarajah D (2012). Advances in the epidemiology, pathogenesis and management of diabetic peripheral neuropathy. Diabetes Metab Res Rev.

[88] Tang W, Shi YQ, Zou JJ (2011). Serum biomarker of diabetic peripheral neuropathy indentified by differential proteomics. Front Biosci (Landmark Ed).

[89] Zhu C, Liang QL, Hu P, Wang YM, Luo GA (2011). Phospholipidomic identification of potential plasma biomarkers associated with type 2 diabetes mellitus and diabetic nephropathy. Talanta.

[90] Glauber H, Brown J (1994). Impact of cardiovascular disease on health care utilization in a defined diabetic population. J Clin Epidemiol.

[91] Park C, Guallar E, Linton JA (2013). Fasting glucose level and the risk of incident atherosclerotic cardiovascular diseases. Diabetes Care.

[92] Nathan DM, Cleary PA, Backlund JY (2005). Intensive diabetes treatment and cardiovascular disease in patients with type 1 diabetes. N Engl J Med.

[93] Gaede P, Vedel P, Larsen N, Jensen GV, Parving HH, Pedersen O (2003). Multifactorial intervention and cardiovascular disease in patients with type 2 diabetes. N Engl J Med.

[94] Voora D, Shah SH, Spasojevic I (2009). The SLCO1B1*5 genetic variant is associated with statin-induced side effects. J Am Coll Cardiol.

[95] Haffner SM, Lehto S, Rönnemaa T, Pyörälä K, Laakso M (1998). Mortality from coronary heart disease in subjects with type 2 diabetes and in nondiabetic subjects with and without prior myocardial infarction. N Engl J Med.

[96] van Dieren S, Beulens JW, Kengne AP (2012). Prediction models for the risk of cardiovascular disease in patients with type 2 diabetes: a systematic review. Heart.

[97] Tanaka S, Tanaka S, Iimuro S (2013). Predicting macro- and microvascular complications in type 2 diabetes: the Japan Diabetes Complications Study/the Japanese Elderly Diabetes Intervention Trial risk engine. Diabetes Care.

[98] Selvin E, Halushka N, Rawlings A (2013). sRAGE and risk of diabetes, cardiovascular disease and death. Diabetes.

[99] Astrup AS (2011). Cardiovascular morbidity and mortality in diabetes mellitus: prediction and prognosis. Dan Med Bull.

[100] Vardi M, Blum S, Levy AP (2012). Haptoglobin genotype and cardiovascular outcomes in diabetes mellitus - natural history of the disease and the effect of vitamin E treatment. Meta-analysis of the medical literature. Eur J Intern Med.

[101] Schunkert H, König IR, Kathiresan S (2011). Large-scale association analysis identifies 13 new susceptibility loci for coronary artery disease. Nat Genet.

[102] Qi Q, Meigs JB, Rexrode KM, Hu FB, Qi L (2013). Diabetes genetic predisposition score and cardiovascular complications among patients with type 2 diabetes. Diabetes Care.

[103] Hernesniemi JA, Seppälä I, Lyytikäinen LP (2012). Genetic profiling using genome-wide significant coronary artery disease risk variants does not improve the prediction of subclinical atherosclerosis: the Cardiovascular Risk in Young Finns Study, the Bogalusa Heart Study and the Health 2000 Survey--a meta-analysis of three independent studies. PLoS One.

[104] Maouche S, Schunkert H (2012). Strategies beyond genome-wide association studies for atherosclerosis. Arterioscler Thromb Vasc Biol.

[105] Davidsson P, Hulthe J, Fagerberg B (2005). A proteomic study of the apolipoproteins in LDL subclasses in patients with the metabolic syndrome and type 2 diabetes. J Lipid Res.

[106] Graff M, Gordon-Larsen P, Lim U (2013). The influence of obesity-related single nucleotide polymorphisms on BMI across the life course: the PAGE study. Diabetes.

[107] Rampersaud E, Mitchell BD, Pollin TI (2008). Physical activity and the association of common FTO gene variants with body mass index and obesity. Arch Intern Med.

[108] Zhang X, Qi Q, Zhang C, Hu FB, Sacks FM, Qi L (2012). FTO genotype and 2-year change in body composition and fat distribution in response to weight-loss diets: the POUNDS LOST Trial. Diabetes.

[109] Mattei J, Qi Q, Hu FB, Sacks FM, Qi L (2012). TCF7L2 genetic variants modulate the effect of dietary fat intake on changes in body composition during a weight-loss intervention. Am J Clin Nutr.

[110] Grant RW, O’Brien KE, Waxler JL (2013). Personalized genetic risk counseling to motivate diabetes prevention: a randomized trial. Diabetes Care.

[111] De Moor MH, Liu YJ, Boomsma DI (2009). Genome-wide association study of exercise behavior in Dutch and American adults. Med Sci Sports Exerc.

[112] Orkunoglu-Suer FE, Gordish-Dressman H, Clarkson PM (2008). INSIG2 gene polymorphism is associated with increased subcutaneous fat in women and poor response to resistance training in men. BMC Med Genet.

[113] Harmon BT, Orkunoglu-Suer EF, Adham K (2010). CCL2 and CCR2 variants are associated with skeletal muscle strength and change in strength with resistance training. J Appl Physiol.

[114] Buford TW, Pahor M (2012). Making preventive medicine more personalized: implications for exercise-related research. Prev Med.

[115] Brownson RC, Boehmer TK, Luke DA (2005). Declining rates of physical activity in the United States: what are the contributors?. Annu Rev Public Health.

[116] National Human Genome Research Institute Frequently asked questions about genetic and genomic science.

[117] Feero WG, Guttmacher AE, Collins FS (2010). Genomic medicine--an updated primer. N Engl J Med.

[118] Bioinformatics Dictionary.com The American Heritage® Science Dictionary. Houghton Mifflin Company. Available at: http://dictionary.reference.com/browse/bioinformatics. Accessed January 16, 2013.

